# A Comparison of the Efficacy and Tolerability of Quinolone-Based Triple Therapy, Quadruple Therapy, and High-Dose Amoxicillin Therapy in *Helicobacter pylori* Eradication

**DOI:** 10.3390/jcm15072480

**Published:** 2026-03-24

**Authors:** Halil Atasoy, Akif Seyrekli, Emre Selim, Aziz Gumuş, Caglayan Keklikkiran, Recep Bedir, Remzi Adnan Akdogan

**Affiliations:** 1 Department of Gastroenterology, Recep Tayyip Erdogan University, Rize 53020, Turkey; akif55_93@hotmail.com (A.S.); emreselim3721@gmail.com (E.S.); c.keklikkiran@edrogan.edu.tr (C.K.); remzi.akdogan@erdogan.edu.tr (R.A.A.); 2 Department of Pulmonary Diseases, Recep Tayyip Erdogan University, Rize 53020, Turkey; aziz.gumus@erdogan.edu.tr; 3 Department of Pathology, Recep Tayyip Erdogan University, Rize 53020, Turkey; bedirrecep@gmail.com

**Keywords:** *Helicobacter pylori*, eradication, efficacy and tolerability, amoxicillin/clavulanic acid

## Abstract

**Objectives**: We aimed to compare the treatment success and patient compliance of triple therapy with levofloxacin, high-dose dual therapy with amoxicillin, and quadruple therapy with bismuth and levofloxacin as first-line treatment for *Helicobacter pylori* (*H. pylori*) eradication using amoxicillin 875 mg + clavulanic acid 125 mg instead of amoxicillin 1 g. **Methods**: Patients who tested positive for *Helicobacter pylori* in the histopathological examination of biopsies taken during upper gastrointestinal endoscopy were initially divided into three different treatment groups. There were 179 patients in the first group, 178 patients in the second group, and 182 patients in the third group. A total of 480 patients from these groups who came for follow-up were included in this study, with 160 patients in each group receiving one of three different treatment protocols. The first group received treatment with amoxicillin 875 mg + 125 mg clavulanic acid twice daily, levofloxacin 500 mg once daily, and pantoprazole 40 mg twice daily (Group 1). The second group received treatment with amoxicillin 875 mg + 125 mg clavulanic acid three times a day, along with pantoprazole 40 mg twice daily (Group 2) as treatment. The third group received treatment with amoxicillin 875 mg + 125 mg clavulanic acid, twice daily, levofloxacin 500 mg once daily, pantoprazole 40 mg twice daily, and bismuth subsalicylate 262 mg2 pieces four times a day (Group 3). *H. pylori* was checked with a stool antigen test 45 days after the 14-day treatment. The groups were compared in terms of treatment success and treatment compliance. **Results**: In Group 1, 150 (90.6%) of 160 patients tested negative for an *H. pylori* antigen in stool samples on day 45 after treatment. This rate was 139 (86.9%) in Group 2 and 148 (92.5%) in Group 3. There were no statistically significant differences between the three groups in terms of treatment success (*p* = 0.233). Side effects were observed in 10 (6.2%) patients in Group 1. Side effects were present in nine (5.6%) patients in Group 2. Side effects were observed in 12 (7.5%) patients in Group 3. There was no significant difference between the groups in terms of patient compliance (*p* = 0.786). **Conclusions**: Treatment success and side effects were similar in all three groups, with no statistical difference. The combination of amoxicillin 875 mg + clavulanic acid 125 mg is at least as effective as amoxicillin 1 g alone.

## 1. Introduction

Increasing antibiotic resistance to the treatment of *Helicobacter pylori* reduces treatment success rates. Amoxicillin, tetracycline, or rifabutin, which have low antibiotic resistance, must be included in the treatment protocol. Since amoxicillin is not available as a single agent in our country, we aimed to determine the efficacy and treatment compliance of the amoxicillin–clavulanic acid combination, which we are forced to use, in three different treatment protocols. *Helicobacter pylori* is a Gram-negative, spiral-shaped, flagellated, microaerophilic bacterium. It settles under the mucus layer in the stomach, causing inflammation known as gastritis. Thanks to the urease enzyme it secretes, it can survive in the stomach for many years, protected from stomach acid. In addition to gastritis, it causes peptic ulcer, atrophic gastritis, gastric adenocarcinoma, and Mucosa-Associated Lymphoid Tissue (MALT) lymphoma [1]. It has been classified as a Group 1 carcinogen by the International Agency for Cancer Research (IARC). It has been reported to be the most common bacterium causing cancer [2]. In addition, it plays a role in the etiology of iron deficiency anemia, B12 deficiency, and idiopathic thrombocytopenic purpura (ITP) [3]. Low socioeconomic status, poor hygiene conditions, crowded family environments, and contaminated water facilitate *H. pylori* transmission. Transmission routes can be fecal–oral and oral–oral [4]. Although the prevalence of *H. pylori* in our country varies depending on different measurement methods, it is gradually decreasing. In a study conducted in 2013 using the urea breath test, the prevalence of *H. pylori* was found to be 82.5%, while in a study conducted in 2025 using the antigen test in stool, this rate was found to be 16.7%. In addition, in the gastric endoscopic biopsies we take daily, *H. pylori* is detected as positive in 75 out of every 100 patients. Due to the improving health and hygiene conditions in our country, a decrease in *H. pylori* frequency is observed [5,6,7,8,9]. Helicobacter pylori can cause hyperacidity or hypoacidity depending on its location in the stomach. The recurrence rate after treatment is low. A meta-analysis found an annual global recurrence rate of 4.3%, a reinfection rate of 3.1%, and a relapse rate of 2% [10]. The rates are similar in our country [11]. The test-and-treat rule applies to patients with dyspepsia. Both invasive and non-invasive tests are available for diagnosis. Invasive tests include endoscopic biopsy with pathological examination, rapid urease test, culture, and molecular tests. Non-invasive tests include the urea breath test, stool antigen test, and serological tests [12]. Serological tests are not routinely recommended as they do not indicate treatment success. *H. pylori* eradication reduces the risk of developing gastric cancer. Treatment involves the combined use of proton pump inhibitors (PPIs) and antibiotics. Increasing antibiotic resistance negatively affects treatment success [13]. Proton pump inhibitors show a synergistic effect with antibiotics. They increase the effectiveness of antibiotics by reducing stomach acidity. Potassium-competitive acid blockers (P-CABs) are used for eradication in combination with antibiotics. Numerous antibiotics (clarithromycin, metronidazole, amoxicillin, tetracycline, furazolidone, rifaximin, levofloxacin, moxifloxacin, sitafloxacin, tinidazole, and rifabutin) and bismuth salts are used in treatment. The Maastricht VI consensus report recommends not using clarithromycin if resistance is >15%. Since clarithromycin resistance is around 30% in our country, it is not used. Resistance to metronidazole and quinolones also increases with clarithromycin. Since single-antibiotic protocols have failed, combination treatment protocols have been developed. Therefore, one of the antibiotics with very low resistance, such as amoxicillin, tetracycline, and rifabutin, must be included in the treatment protocols. Clavulanic acid is a beta-lactamase inhibitor that prevents the development of resistance when used with penicillins. Amoxicillin is a good option except for those with a penicillin allergy. When used at high doses, it can be administered as a single antibiotic together with a PPI. Tetracycline is not preferred due to its tendency to cause esophagitis and difficulty in use. Rifabutin is not available in our country. Amoxicillin alone has probably not yet been withdrawn in our country because it is inexpensive. Therefore, we use the combination of amoxicillin 875 mg + clavulanic acid 125 mg for *H. pylori* eradication. We aimed to investigate the efficacy of this combination in three different treatment protocols.

## 2. Materials and Methods

Three different treatment groups were initially formed of patients who tested positive for *Helicobacter pylori* in the histopathological examination of biopsies taken during upper gastrointestinal endoscopy. Patients who presented to the gastroenterology outpatient clinic of Recep Tayyip Erdogan University in 2025 were included in the study. There were 179 patients in the first group, 178 patients in the second group, and 182 patients in the third group. A total of 480 patients were included in the study, with 160 patients randomly assigned to each of the three different treatment protocols from among those who came for follow-up in these groups. The study design is shown in Table 1.

Our study is a prospective observational study. The first group received treatment with amoxicillin 875 mg + 125 mg clavulanic acid twice daily, levofloxacin 500 mg once daily, and pantoprazole 40 mg twice daily (Group 1). The second group received treatment with amoxicillin 875 mg + 125 clavulanic acid three times a day and pantoprazole 40 mg twice daily (Group 2) treatment. The third group received treatment with amoxicillin 875 mg + 125 mg clavulanic acid twice daily, levofloxacin 500 mg once daily, pantoprazole 40 mg twice daily, and bismuth subsalicylate 262 mg at two pieces four times a day (Group 3). The treatment duration was set at 14 days. Patients aged 22 to 75 years who had *H. pylori* detected in endoscopically performed gastric biopsies were included in this study. Patients who used the treatment for less than ten days were excluded from this study.

After completing treatment, participants were instructed not to use PPIs for the last 15 days. Forty-five days later, a fecal antigen test (Rapid Cassette Test (Feces) (Acro, Biotech, Montclair, WI, USA) was performed to detect *H. pylori*. Those who tested negative were considered treated, while those who tested positive were considered untreated. Additionally, patient compliance with treatment was determined among those who continued treatment despite mild side effects (diarrhea, dyspepsia, skin redness, nausea). Those who discontinued treatment within ten days of initiation due to side effects were excluded from the study. Ethical committee approval was obtained from Recep Tayyip Erdogan University Non-Interventional Clinical Research Ethics Committee Presidency under number 2024/313 and E-40465587-050.01.04-1300. The three groups were compared in terms of treatment success and side effects.

Statistical evaluations were performed using the IBM-SPSS program (SPSS version 27; SPSS Inc., Chicago, IL, USA). A power analysis was conducted. It was determined that at least 400 patients should be included in the study.

The normality of continuous variables was examined using the Kolmogorov–Smirnov test. Continuous variables were presented as the mean ± standard deviation, and categorical variables were presented as number (%). The comparison of the three groups was performed using the ANOVA test. The comparison of categorical variables was performed using the Chi-square test. A *p*-value < 0.05 was considered statistically significant. Statistical analysis was performed per protocol on those who participated in and completed the study.

## 3. Results

All three groups were similar in terms of endoscopic diagnosis (*p* = 0.91). Histopathological diagnoses were reported as active chronic gastritis, chronic gastritis, and ulceration. No significant difference was found between the three groups in terms of histopathological diagnosis. (*p* = 0.135) All patients who were found to be normal endoscopically were diagnosed with chronic gastritis histopathologically. The findings are shown in Table 2. In Group 1, *H. pylori* was negative in 150 (90.6%) of 160 patients tested via the antigen stool test on day 45 after treatment. This rate was 139 (86.9%) in Group 2 and 148 (92.5%) in Group 3. There was no statistically significant difference in treatment success between the three groups (*p* = 0.233).

Side effects were observed in 10 (6.2%) patients in Group 1. Three of these patients reported mild diarrhea, four reported abdominal pain, two reported nausea, and one reported itching. Nine (5.6%) patients in Group 2 reported side effects. Three of these patients reported abdominal pain, two reported diarrhea, two reported nausea, and two reported itching. Side effects were observed in 12 (7.5%) patients in Group 3. Three of these patients reported nausea, three reported abdominal pain, two reported constipation, two reported itching, and two reported bloating. There were no significant differences in side effects or patient compliance among the three groups (*p* = 0.786). The findings are shown in Table 3.

## 4. Discussion

Although the prevalence of *H. pylori* is gradually decreasing in our country, the figures on this subject are contradictory. Results vary depending on the method used to detect *H. pylori*. The *H. pylori* rate detected in our endoscopically obtained biopsies was 75%, while the *H. pylori* positivity rate detected by the urea breath test was 82.5% [6]. The rate detected by the fecal antigen test was 16.7% in adults [5]. Although the sensitivity of the antigen test in stool is reported as 94% in the literature [14], there is a possibility of a high false negative rate in our results. From this perspective, this rate is estimated to be around 50%. Due to increasing antibiotic resistance, the success rate of *H. pylori* eradication therapy is decreasing. According to the Maastricht VI consensus report, a success rate of over 80% is required for *H. pylori* treatment to be considered effective [1]. Unsuccessful treatment necessitates second- and third-line treatments, significantly increasing treatment costs. To achieve this, antibiotics with low resistance rates must be used as a basis. We have three antibiotics with low resistance rates at our disposal. These are amoxicillin, tetracycline, and rifabutin. In our country, probably because it is inexpensive, amoxicillin preparations are not available alone, so we had to use the combination of amoxicillin 875 mg + clavulanic acid 125 mg for *H. pylori* treatment. We could not find any studies in the literature demonstrating the efficacy of this combination. We designed this study to evaluate the effectiveness of treatment based on treatment outcomes. Since clarithromycin resistance is over 30% in our country, it is not used in accordance with Maastricht VI recommendations. Metronidazole resistance is also high. Resistance to quinolone antibiotics is also increasing. In a meta-analysis of 2660 samples covering the years 2011–2021, metronidazole resistance was found to be 42.1%, levofloxacin 37.6%, clarithromycin 31.5%, amoxicillin 2.6%, tetracycline 0.87%, and rifabutin 0.17% [13]. Another study reported that clarithromycin, metronidazole, and levofloxacin resistance rates were greater than 15% [15]. Resistance rates in our country were found to be 36.7% for clarithromycin, 35.5% for metronidazole, 29.5% for levofloxacin, 2.9% for amoxicillin, and 1% for tetracycline [16]. Bismuth salts are used in *H. pylori* eradication due to their bactericidal effects and low rate of side effects. However, daily multiple dosing makes treatment compliance difficult. A single-capsule bismuth preparation is not available in our country. However, the treatment discontinuation rate due to side effects is around 1.7% [17]. In our results, the side effect rate was 6.2% in Group 1, 5.6% in Group 2, and 7.5% in Group 3. These rates were determined among those who completed treatment despite side effects. Participants who discontinued treatment due to side effects were excluded from the study because we could not measure treatment success in these cases. Side effects included abdominal pain, nausea, diarrhea, and constipation in the bismuth group. Combining amoxicillin and tetracycline, two antibiotics with low resistance, may increase efficacy, but it is not practical in clinical practice. Pantoprazole was preferred as the PPI. In our study, all three groups received the treatment protocol as first-line therapy, which included amoxicillin 875 mg + clavulanic acid 125 mg. Age, gender, and endoscopic diagnoses had no effect on treatment success. Histopathological diagnoses were reported as active chronic gastritis, chronic gastritis, and ulceration. No significant difference was found between the three groups in terms of histopathological diagnosis (*p* = 0.135). All patients who were found to be normal endoscopically were diagnosed with chronic gastritis histopathologically. While levofloxacin-based triple therapy (Group 1) has shown a treatment success rate of 80–90% in various studies [17,18,19,20,21,22,23], it was found to be 90.6% in our study. The additional contribution of the amoxicillin + clavulanic acid combination, the low local antibiotic resistance, and the high false positivity of the stool antigen test may have contributed to this high result. The success rate of dual therapy with high-dose amoxicillin + clavulanic acid (3 g daily) was 86.9%. Although this was lower than in Group 1, it was not statistically significant. Skipping doses may be a problem encountered in this treatment. Another factor is that single-antibiotic regimens have lower success rates. We preferred levofloxacin instead of metronidazole in the quadruple therapy. The success rate of treatment with bismuth-containing levofloxacin was found to be 92.5%. All these findings indicate that treatment regimens containing amoxicillin + clavulanic acid are not inferior to regimens containing amoxicillin alone. A study conducted in 2014 found a 92% success rate with levofloxacin-based treatment. This rate was also found to be 91.8% with moxifloxacin [18]. In our study, the results were similar when compared to amoxicillin supplemented with clavulanic acid. In a meta-analysis covering 4574 patients, the success rate of levofloxacin treatment was found to be 80.6% [19]. The 2024 American College of Gastroenterology (ACG) guidelines recommend levofloxacin for second-line or rescue therapy based on antibiotic susceptibility testing [20]. However, in daily practice, it is also used as first-line therapy in areas where clarithromycin is not available due to limited options. Again, rifabutin triple therapy or vanazapan dual therapy are recommended as alternatives in the second line of treatment. Bismuth quadruple therapy is also recommended in cases of penicillin allergy [21,22,23]. There are publications reporting that competitive acid blockers are at least as effective as PPIs in *H. pylori* eradication therapy [24,25]. However, since these drugs are not available in our country, we used PPIs. In cases of treatment failure, antibiotic susceptibility testing can be performed using molecular tests rather than cultures. However, the use of these tests is not yet widespread. Studies comparing the success of empirical treatment with sensitivity-based treatments have failed to demonstrate the superiority of sensitivity-based treatment in either first-line treatment or rescue therapy. In cases of treatment failure, secondary resistance to antibiotics has been found to be low with levofloxacin. High secondary resistance is more common due to prior use of these antibiotics for other reasons [26,27].

There are older studies comparing amoxicillin in combination with clavulanic acid in *H. pylori* eradication. Some of these studies reported that adding clavulanic acid increased treatment success [28,29].

There have been previous studies comparing amoxicillin in combination with clavulanic acid for *H. pylori* eradication. Some of these studies reported that adding clavulanic acid increased treatment success. However, a study by Crispino P et al. reported that adding clavulanic acid did not affect the *H. pylori* eradication rate [30]. These findings suggest that using clavulanic acid in combination with amoxicillin is a good option when amoxicillin alone is unavailable.

The limitation of our study was the possibility of false negatives in the stool antigen test results, although the sensitivity was reported as 94%. Moreover, there is no complete agreement between histological testing and the urea breath test. Since re-checking for the presence of *H. pylori* via endoscopic biopsy after treatment places an additional burden on both physicians and patients, it is impractical for clinical use.

## 5. Conclusions

In first-line treatment, where we used the combination of amoxicillin 875 mg + clavulanic acid 125 mg instead of amoxicillin 1 g, three different treatment protocols were compared in terms of treatment success and patient compliance. In Group 1, which included amoxicillin 875 mg + clavulanic acid 125 mg twice daily, levofloxacin 500 mg once daily, and pantoprazole 40 mg twice daily, treatment success was 90.6%, and side effects were reported at a rate of 6.2%. In Group 2, which included amoxicillin 875 mg + clavulanic acid 125 mg three times a day and pantoprazole 40 mg twice daily, treatment success was 86.9% and side effects were reported at a rate of 5.6%. In Group 3, which included amoxicillin 875 mg + clavulanic acid 125 mg twice daily, levofloxacin 500 mg once daily, pantoprazole 40 mg twice daily, and bismuth subsalicylate 262 mg at two pieces four times a day, treatment success was 92.5% and side effects were reported at a rate of 7.5%. Treatment success and side effects were similar across all three groups, with no statistically significant differences. The combination of amoxicillin 875 mg + clavulanic acid 125 mg is at least as effective as amoxicillin 1 g alone.

## Figures and Tables

**Table 1:** Study design.

	Group 1	Group 2	Group 3
Started the study	*n*: 179	*n*: 178	*n*: 182
Completed the study	*n*: 160	*n*: 160	*n*: 160

**Table 2:** Demographic and baseline characteristics of treatment groups.

	Group 1 (*n*: 160)	Group 2 (*n*: 160)	Group 3 (*n*: 160)	*p* Value
Age	48 ± 13	51 ± 14	50 ± 12	0.126
Gender (Female/Male)	102/58	93/67	86/74	0.191
**Endoscopic diagnosis**				0.091
Normal	12 (7.5%)	13 (8.1%)	15 (9.4%)	
Gastritis	135 (84.4%)	119 (74.4%)	129 (80.6%)	
Ulcer	13 (8.1%)	28 (17.5%)	16 (10%)	
**Histopathological** **diagnosis**				0.135
Active chronic gastritis	66 (41.3%)	73 (45.6%)	62 (38.8%)	
Chronic gastritis	90 (56.2%)	84 (52.5%)	93 (58.1%)	
Ulceration	4 (2.5%)	3 (1.9%)	5 (3.1%)	

**Table 3:** Treatment success and side effect rates of groups.

	Group 1 (*n*: 160)	Group 2 (*n*: 160)	Group 3 (*n*: 160)	*p* Value
Treatment compliance (Neg/Pos)	10/150 (6.2%)	9/151 (5.6%)	12/148 (7.5%)	0.786
Treatment success (Neg/Pos)	145/15 (90.6%)	139/21 (86.9%)	148/12 (92.5%)	0.233

Neg: negative; Pos: positive.

## Data Availability

The datasets used and/or analyzed during the current study are available from the corresponding author on reasonable request.
